# Effectiveness of a WeChat-based personalized rehabilitation nursing model for urinary incontinence after prostatectomy: a retrospective cohort study

**DOI:** 10.3389/fonc.2025.1617035

**Published:** 2025-11-21

**Authors:** Sijin Chen, Ruochen Zhang, Zijun Zou, Yaojing Yang, Le Lin, Liefu Ye, Jinfeng Wu, Yuanman Zhang, Wei Song, Qichen Wei, Yongbao Wei

**Affiliations:** 1 Department of Urology, Hunan Provincial People’s Hospital, The First Affiliated Hospital of Hunan Normal University, Changsha, Hunan, China; 2 Shengli Clinical Medical College of Fujian Medical University, Department of Urology, Fujian Provincial Hospital, Fuzhou University Affiliated Provincial Hospital, Fuzhou, China; 3 Department of Urology, Guizhou Provincial People’s Hospital, Guiyang, China; 4 Department of Urology, Gutian County Hospital, Ningde, China

**Keywords:** personalized rehabilitation nursing model, urinary incontinence, prostatectomy, retrospective cohort study, prostate cancer

## Abstract

Postoperative urinary incontinence (UI) is one of the most common complications affecting the quality of life in prostate cancer patients. Traditional nursing models mainly rely on outpatient guidance, making it difficult to provide continuous and dynamic rehabilitation support, and patient compliance is generally low. A personalized nursing model based on the WeChat platform offers a new strategy for managing postoperative UI through real-time interaction, training delivery, and individualized feedback. This study enrolled 123 patients with postoperative UI after radical prostatectomy, divided into a WeChat education group (n=65) and a traditional nursing group (n=58). Baseline characteristics, including UI severity and quality of life scores, were comparable between groups. After six months of follow-up, the WeChat group showed a 120 mL reduction in 24-hour urine leakage, an average decrease of 5.8 points in ICIQ-SF score, and an improvement of 0.18 in EQ-5D score—all significantly better than the traditional group (P<0.01). The WeChat group also demonstrated higher compliance (92%) and nursing satisfaction scores (9.2/10) than the traditional group (P<0.01). Multivariate linear regression analysis identified compliance, initial urine leakage volume, and age as independent predictors of UI improvement, while surgical approach and lymph node dissection were not significantly associated. These findings suggest that the WeChat-based nursing model effectively improves postoperative urinary continence and quality of life, offering a scalable, efficient, and individualized digital nursing strategy for managing UI after prostatectomy.

## Background

Prostate cancer is among the most prevalent malignancies in men worldwide. Treatment modalities include radical prostatectomy, radiotherapy, hormone therapy, and chemotherapy (4). While surgical removal effectively eliminates the primary tumor and prolongs survival, postoperative urinary incontinence remains a major complication (4). This functional disorder not only affects physical health but also imposes psychological distress and reduced quality of life (20, 27). Studies indicate that 20–30% of patients experience persistent UI even one year after surgery, with some enduring symptoms for years (3, 23).

Pelvic floor muscle training (PFMT) is a key method for improving UI by restoring urethral sphincter and pelvic floor muscle function (26). However, traditional care models primarily rely on face-to-face medical guidance and outpatient follow-ups, which often fail to deliver continuous and personalized support (17). Frequent follow-up visits also pose financial and logistical burdens, especially for patients in rural or economically disadvantaged areas (6). Furthermore, low compliance and reduced motivation limit the effectiveness of such care. Smoking has been reported to impair pelvic floor muscle function and delay postoperative recovery by reducing tissue oxygenation and microvascular perfusion, thereby increasing the risk of urinary incontinence. Consequently, evaluating compliance among smokers is clinically relevant, as adherence to rehabilitation programs may have a greater impact on outcomes in this subgroup.

With the growing adoption of internet and mobile technologies, WeChat-based health management has emerged as a feasible alternative. As one of the most widely used social platforms in China, WeChat (developed by Tencent Holdings Ltd., Shenzhen, China) offers instant communication and supports health education through subscription accounts, chat groups, and mini-programs. It is a commercially operated but free-to-use application that allows healthcare providers to communicate with patients without additional costs. In this study, nurses utilized existing WeChat functions to deliver postoperative instructions, PFMT videos, and personalized rehabilitation plans while monitoring patient responses in real time. This model transcends the limitations of traditional care, efficiently reaching patients with UI and enhancing engagement and adherence.

Although WeChat-based nursing has been increasingly applied in chronic disease management (25) and postoperative rehabilitation, systematic research specifically targeting UI after prostatectomy is still limited. Most existing studies report preliminary results or small-scale trials without long-term evaluations (12, 13, 29). Furthermore, few studies have established data-driven, dynamically adaptive WeChat models that quantify nursing engagement and link behavioral indicators to recovery outcomes. This study therefore retrospectively analyzes the application of WeChat education in prostate cancer patients with postoperative UI, assess its long-term effects, identify key factors influencing UI improvement, and provide scientific evidence for optimizing WeChat-based personalized care. The results may accelerate recovery of urinary continence and guide modern nursing practice in the digital era.

## Methods

### Study design

This retrospective cohort study evaluated the long-term effectiveness of a WeChat-based personalized nursing model in prostate cancer patients with postoperative UI. Patients were allocated to the WeChat education or traditional care group. This retrospective design analyzed patients who had already received postoperative care via either the WeChat program or traditional outpatient follow-up between January 2020 and December 2023. The groups were not randomized but were matched for baseline demographic and clinical characteristics (age, BMI, comorbidities, surgical approach, and initial incontinence severity) to minimize selection bias and confounding. Outcomes included UI improvement, compliance, and quality of life. Eligible patients underwent surgery between January 2020 and December 2023. The study was approved by the institutional ethics committee. Patient data were anonymized and collected per medical ethics standards to ensure privacy and confidentiality.

### Participants

Inclusion criteria: confirmed UI after radical prostatectomy, minimum of six months of WeChat-based or conventional care, complete clinical records, and consent to participate.

Exclusion criteria: concomitant urological conditions (e.g., neurogenic bladder, severe urethral stricture), incomplete data or insufficient follow-up, cognitive impairment, or severe psychiatric disorders.

### Data collection

Data were collected from electronic medical records and the WeChat education platform, including demographics (age, gender, BMI), clinical characteristics (preoperative staging, comorbidities), and outcomes (International Consultation on Incontinence Questionnaire-Short Form (ICIQ-SF) was used to evaluate the severity and impact of urinary incontinence (score range 0–21; higher scores indicate worse symptoms) (14), the 24-hour pad test quantified urine leakage volume (mL) (8), EuroQol Five-Dimension Questionnaire (EQ-5D) was used to assess overall health-related quality of life (index range 0–1, with higher scores indicating better well-being) (8), while the 36-Item Short Form Health Survey (SF-36) measured physical and mental health domains (score range 0–100) (10)). Nursing metrics included the completion rate, defined as the proportion of scheduled WeChat-based rehabilitation activities—such as daily PFMT video sessions, health education modules, and feedback submissions—that each participant completed and recorded in the platform.

This metric was automatically generated by the WeChat system based on task completion logs, and reviewed by the nursing staff for data accuracy rather than subjective evaluation. The feedback frequency was defined as the average number of interactive communications (messages, questions, or uploads) per patient per week.

### Group allocation and care models

WeChat group patients received personalized education and rehabilitation via WeChat groups, mini-programs, or official accounts. Daily content included PFMT videos, customized plans, and Q&A sessions. Nurses adjusted protocols based on patient feedback. Each participant in the WeChat group received continuous nursing guidance for six months after catheter removal. Educational and rehabilitation content was delivered daily via WeChat mini-program modules, including 3–5 minute PFMT videos, reminders, and weekly summaries. Nurses responded to patient inquiries in real time and followed a standardized process of assessment, goal setting, training instruction, feedback review, and individualized plan adjustment. Completion rate and feedback frequency were objectively recorded by the system and verified by the nursing team to ensure fidelity and reproducibility. The traditional group received routine postoperative care and outpatient PFMT guidance without WeChat support. The personalized WeChat education program consisted of three major modules: (1) Daily functional training module, delivering 5–10-minute PFMT videos and reminders tailored to patient progress; (2) Health education module, including weekly lectures and infographics covering bladder function recovery, lifestyle adjustment, and medication adherence; and (3) Interactive feedback module, allowing patients to upload training logs and communicate directly with nurses through private messages or group Q&A. Nurses reviewed feedback twice weekly and dynamically adjusted training intensity and frequency based on patient-reported progress.

### Statistical analysis

Baseline characteristics were summarized descriptively. Differences in outcomes were tested using independent-sample t-tests or Mann-Whitney U tests. Categorical variables were analyzed using chi-square tests. Multivariate linear regression identified key factors affecting UI improvement, with trend analysis used to assess long-term effects. All statistical tests were two-sided, and a *P* value < 0.05 was considered statistically significant. Although no *a priori* power calculation was performed due to the retrospective design, a *post-hoc* analysis based on the observed difference in ICIQ-SF score reduction (Δ = 5.8 ± 3.1 vs 3.2 ± 3.0, P < 0.01) indicated a statistical power greater than 0.90 at α = 0.05, confirming that the sample size (n = 123) was adequate to detect clinically meaningful effects.

## Results

### Patient inclusion and baseline characteristics

A total of 123 patients were included: 65 in the WeChat group and 58 in the traditional group. Patients were excluded due to incomplete data, or insufficient follow-up. There were no significant differences between groups in age, BMI, PSA levels, pathological stage, Gleason score, neoadjuvant therapy, comorbidities, surgical approach, NVB preservation, LUTS history, or baseline ICIQ-SF scores (P > 0.05), indicating good baseline comparability (**Table 1**).

**Table 1:** Baseline characteristics of patients in the WeChat and traditional nursing groups.

Variable	WeChat group (n=65)	Traditional group (n=58)	P-value
Age (years)	67.2 ± 5.4	66.8 ± 6.0	0.72
BMI (kg/m²)	23.4 ± 2.1	23.1 ± 2.5	0.65
Preoperative PSA (ng/mL)	12.6 ± 4.7	13.1 ± 5.2	0.58
Postoperative PSA (ng/mL)	0.06 ± 0.02	0.07 ± 0.03	0.44
Pathological stage (tagel	28 (43.1%)	26 (44.8%)	0.84
Gleason score ≥c	31 (47.7%)	30 (51.7%)	0.67
Neoadjuvant therapy	12 (18.5%)	11 (19.0%)	0.93
Comorbidities	36 (55.4%)	32 (55.2%)	0.98
Surgical method (robotic)	39 (60.0%)	36 (62.1%)	0.81
Bilateral NVB preservation	40 (61.5%)	36 (62.1%)	0.94
Prior LUTS symptoms	22 (33.8%)	20 (34.5%)	0.92
Baseline ICIQ-SF score	13.2 ± 2.1	13.0 ± 2.3	0.64

### UI improvement

At 6 months, the WeChat group reduced 24-hour urine leakage by 120 ± 40 mL vs 60 ± 30 mL in the traditional group (P < 0.01). ICIQ-SF scores dropped by 5.8 ± 1.2 vs 3.1 ± 1.5 points (P < 0.01). The UI improvement rates were 85% vs 70%, with 40% of the WeChat group achieving dryness vs 25% in the traditional group (P = 0.03) (**Table 2**). Among patients with bilateral NVB preservation, the improvement rate was 90% vs 75% without NVB preservation (P = 0.04).

**Table 2:** Urinary incontinence outcomes at 6 months.

Outcome	WeChat group (n=65)	Traditional group (n=58)	P-value
24h urine leakage reduction (mL)	120 ± 40	60 ± 30	<0.01
ICIQ-SF score improvement	5.8 ± 1.2 (95% CI 5.5–6.1)	3.1 ± 1.5 (95% CI 2.7–3.5)	<0.01
Improvement rate (%)	85.00%	70.00%	<0.01
Complete dryness rate (%)	40.00%	25.00%	0.03
Improvement with bilateral NVB	90.00%	75.00%	0.04

Values are mean ± SD unless otherwise stated.

Between-group differences in ICIQ-SF reduction exceeded the *minimal clinically important difference* (MCID ≥ 2 points), indicating clinically meaningful improvement.

### Quality of life

EQ-5D scores improved from 0.62 ± 0.08 to 0.80 ± 0.07 in the WeChat group, and from 0.61 ± 0.09 to 0.73 ± 0.08 in the traditional group (P = 0.02). SF-36 physical and mental dimensions improved by 15.6 ± 3.8 and 12.3 ± 3.1 points, respectively, in the WeChat group, indicating superior functional recovery compared with traditional group (8.2 ± 3.5 and 6.5 ± 2.8, P < 0.01) (**Table 3**). Subgroup analysis showed greater QoL improvement in smokers (10.5 vs 6.0, P = 0.03), lymph node dissection patients (14.0 vs 9.5, P = 0.04), and those receiving adjunctive medication (12.0 vs 7.0, P = 0.02).

**Table 3:** Quality of life improvement.

Variable	WeChat group	Traditional group	P-value
EQ-5D score improvement	0.18 ± 0.06 (95% CI 0.16–0.20)	0.12 ± 0.05 (95% CI 0.10–0.14)	0.02
SF-36 Physical Health improvement	15.6 ± 3.8	8.2 ± 3.5	<0.01
SF-36 Mental Health improvement	12.3 ± 3.1	6.5 ± 2.8	<0.01
Smokers’ SF-36 improvement	10.5 ± 2.8	6.0 ± 2.5	0.03
LN dissection subgroup improvement	14.0 ± 3.2	9.5 ± 2.7	0.04
Adjunctive therapy subgroup	12.0 ± 3.0	7.0 ± 2.5	0.02

Δ values denote mean change ± SD from baseline.

EQ-5D = EuroQol Five-Dimension Questionnaire; SF-36 = 36-Item Short Form Health Survey.

EQ-5D improvement (Δ 0.06) exceeded the MCID (≥ 0.05), demonstrating clinically meaningful QoL benefit.

### Compliance and engagement

The WeChat group achieved a **92% completion rate**, completed and recorded through the WeChat platform, compared with 68% in the traditional group (P < 0.01).

All activity data were automatically tracked by the system, and nursing staff verified completion records for accuracy rather than by subjective assessment.

Weekly feedback frequency averaged 4.8 vs 2.5 interactions per patient (P < 0.01), and satisfaction scores were 9.2 ± 0.8 vs 7.6 ± 1.1 (P < 0.01) (**Table 4**).

**Table 4:** Nursing compliance and patient engagement.

Variable	WeChat group (n=65)	Traditional group (n=58)	P-value
Rehabilitation plan completion rate	92%	68%	<0.01
Average weekly feedback frequency	4.8	2.5	<0.01
Satisfaction score (out of 10)	9.2 ± 0.8	7.6 ± 1.1	<0.01
Compliance in smokers	85%	60%	<0.01
Compliance with LN dissection	90%	70%	0.04

Completion rate = proportion of scheduled WeChat-based rehabilitation activities completed and logged in the platform (system-tracked data, verified by nursing staff).

Feedback frequency = average number of interactive communications (messages, uploads, or responses) per patient per week.

Among smokers, compliance was 85% vs 60%; in lymph node dissection patients, it was 90% vs 70% (P < 0.01 and P = 0.04, respectively).

### Rehabilitation speed

The average time to continence improvement was 6.4 ± 1.6 weeks vs 9.2 ± 2.0 weeks (P < 0.05). Within 3 months, 78% of patients in the WeChat group experienced marked improvement compared with 46% in traditional care (P < 0.05), demonstrating faster functional recovery. Dryness was also achieved significantly faster (**Table 5**).

**Table 5:** Recovery speed.

Variable	WeChat Group (n=65)	Traditional Group (n=58)	P-value
Time to continence improvement (weeks)	6.4 ± 1.6	9.2 ± 2.0	<0.05
Improvement within 3 months (%)	78%	46%	<0.05
Time to achieve complete dryness (weeks)	9.8 ± 2.3	13.6 ± 2.7	<0.01

Time to continence improvement = weeks from surgery to first documented ≥ 50% reduction in leakage volume.

All comparisons statistically and clinically significant (*P* < 0.05).

### Influencing factors

Multivariate regression identified compliance (β = 0.85, P < 0.01), initial leakage volume (β = 0.56, P < 0.01), age (β = -0.34, P < 0.05), NVB preservation (β = 0.20, P = 0.04), and adjunctive therapy (β = 0.15, P = 0.03) as independent predictors of UI improvement. Surgical approach (robotic vs laparoscopic, β = 0.12, P = 0.15) and lymph node dissection (β = 0.10, P = 0.10) were not significant (**Table 6**).

**Table 6:** Multivariate linear regression for UI improvement.

Variable	β Coefficient	95% Confidence Interval	P-value
Compliance	0.85	0.75 ~ 0.95	<0.01
Initial urinary leakage	0.56	0.40 ~ 0.72	<0.01
Age	-0.34	-0.45 ~ -0.23	<0.05
Preoperative Gleason score	-0.2	-0.35 ~ -0.05	0.04
Preoperative PSA level	0.1	0.03 ~ 0.17	0.01
Number of chronic comorbidities	-0.25	-0.42 ~ -0.08	0.02
Surgical approach (robotic vs. laparoscopic)	0.12	-0.05 ~ 0.29	0.15
Bilateral NVB preservation	0.2	0.08 ~ 0.32	0.04
Lymph node dissection	0.1	-0.03 ~ 0.23	0.1
Adjuvant pharmacotherapy	0.15	0.03 ~ 0.27	0.03

β coefficients with 95% confidence intervals illustrate effect sizes and clinical relevance of each predictor.

## Discussion

The results of this retrospective cohort study underscore the clinical value and feasibility of a WeChat-based personalized rehabilitation nursing model in managing UI after radical prostatectomy. This model demonstrated superior outcomes compared to traditional nursing approaches in terms of symptom improvement, quality of life, patient engagement, and recovery time. Beyond statistical significance, these improvements were clinically meaningful: the mean reduction of 2.7 points in ICIQ-SF and increase of 0.06 in EQ-5D exceeded the minimal clinically important difference (MCID) thresholds (≥2 and ≥0.05, respectively), confirming practical benefits for patients. These findings not only reflect the growing potential of digital health interventions in urological care but also contribute to the evolving paradigm of precision nursing in the postoperative period. In terms of feasibility, the program required no additional equipment or paid applications. The intervention was implemented using existing WeChat functions and nursing manpower, with nurses dedicating fixed daily time slots to review feedback and adjust care plans, which proved sustainable within routine workloads. As a preliminary single-center study, our findings provide early but valuable evidence supporting the feasibility of digital personalized rehabilitation nursing, which warrants validation in larger multicenter randomized controlled trials.

One of the most striking observations was the significantly greater reduction in 24-hour urine leakage and ICIQ-SF scores in the WeChat group, with a concurrent rise in continence recovery rate and quality of life. These improvements can be attributed to several synergistic elements embedded in the digital intervention. First, the integration of daily PFMT videos, individualized rehabilitation plans, and real-time Q&A sessions likely promoted behavioral reinforcement and neuromuscular adaptation (7, 19). Second, the instant feedback mechanism allowed for dynamic adjustments based on patient status, preventing the stagnation or improper execution often seen in traditional outpatient settings (28).

Compliance emerged as a pivotal factor influencing clinical outcomes, supported by both descriptive and multivariate analyses. Patients in the WeChat group demonstrated a 24% higher completion rate and more frequent weekly feedback, reinforcing the notion that active engagement is crucial to sustained functional improvement. These results echo previous findings in chronic disease management where mobile health tools improved adherence and health outcomes (17, 19). Importantly, WeChat’s high accessibility and familiarity among Chinese patients likely contributed to the low technical barrier and strong participation, especially among elderly populations who may otherwise struggle with complex health apps (22).

Interestingly, the benefits of the WeChat model extended beyond physical recovery. Substantial gains were noted in the EQ-5D and SF-36 physical and mental domains, suggesting improvements in both functional capacity and psychological well-being. Postoperative UI is known to induce emotional distress, embarrassment, and social withdrawal (16, 20). The structured guidance and constant reassurance provided through the digital platform likely alleviated anxiety and restored a sense of control, particularly in vulnerable groups such as smokers or those undergoing lymph node dissection (11). These findings underscore the psychosocial dimension of continence rehabilitation and the capacity of digital nursing to provide holistic care.

Among clinical predictors, bilateral nerve-sparing (NVB) surgery was significantly associated with better outcomes, consistent with literature indicating that preservation of neurovascular bundles enhances sphincter integrity and promotes earlier recovery of continence (15). However, surgical approach (robotic vs laparoscopic) and lymph node dissection did not significantly influence continence improvement, suggesting that functional outcomes depend more on individualized postoperative care than on technical variations alone (1, 5, 18). These insights may guide urologists and nurses in tailoring rehabilitation strategies based on intraoperative decisions and patient-specific profiles.

Adjunctive therapies, including medications or supportive interventions, were modest yet statistically significant contributors to UI recovery (9, 24). These likely assist in tissue regeneration or neuromuscular recovery, complementing the behavioral components of PFMT. Further research could explore the optimal combination and timing of such therapies within a digital care framework.

Another noteworthy finding is the accelerated rehabilitation speed in the WeChat group. Patients regained significant continence approximately three weeks earlier than those in the traditional care group, with 78% showing marked improvement within 3 months. Early recovery not only enhances quality of life but may also reduce downstream medical costs and psychological burden, offering public health benefits (2, 21).

The present study is a single-center retrospective cohort analysis, which limits the level of evidence and causal inference. Randomization and blinding were not feasible due to the real-world design; however, baseline matching was applied to mitigate selection bias. Future prospective multicenter randomized controlled trials are warranted to validate and extend these findings. Despite these strengths, several limitations must be acknowledged. The retrospective, single-center design introduces selection bias and limits generalizability. Patients who opted into the WeChat program may have been more health-literate or motivated, potentially skewing compliance and outcomes. Although baseline characteristics were well balanced, unmeasured confounders such as education level or prior digital literacy could influence results. Another limitation is that the sample size was determined by the available cases rather than a pre-study power calculation, which may affect generalizability. However, *post-hoc* analysis suggested sufficient power for the primary outcomes, supporting the reliability of the main findings. Another limitation is that the retrospective dataset lacked information on education, digital literacy, and family support, which may influence patients’ engagement with digital interventions. Future prospective studies should include these variables to further refine model accuracy. Moreover, the follow-up period, while adequate for assessing short- to mid-term effects, may not capture late recurrences of UI or long-term sustainability of digital engagement. Lastly, satisfaction scores, while favorable, were self-reported and may be subject to response bias. Future studies should aim to validate these findings through multicenter, randomized controlled trials with larger sample sizes and stratified analysis. Incorporating wearable devices or AI-assisted feedback into the WeChat platform could further enhance precision and automation. Additionally, expanding the scope of digital nursing to include other complications such as erectile dysfunction, bowel symptoms, or psychosocial distress could offer a more comprehensive postoperative care ecosystem.

## Conclusion

In contrast to earlier descriptive studies, our model integrates quantitative feedback loops and individualized algorithmic adjustments, offering a refined, evidence-based approach to digital rehabilitation nursing. A WeChat-based personalized nursing model significantly improves urinary continence and quality of life after prostatectomy. Key contributors include patient compliance and NVB preservation, with adjunctive therapy offering additional benefits. Compared to traditional care, this model enables more efficient and precise nursing, accelerating recovery and setting a new direction for postoperative UI management and future nursing innovation.

## Data Availability

The raw data supporting the conclusions of this article will be made available by the authors, without undue reservation.
